# Health status of families: A comparative study of one-parent and two-parent families in Ondo State, Nigeria

**DOI:** 10.4102/phcfm.v10i1.1550

**Published:** 2018-07-30

**Authors:** Cecilia B. Bello, Omolola Irinoye, Oluwaseyi A. Akpor

**Affiliations:** 1 Department of Nursing Science, College of Medicine and Health Sciences, Afe Babalola University, Nigeria

## Abstract

**Background:**

The family plays a central role in the provision and maintenance of health status of its members and all factors that contribute to achieving optimal health.

**Aim:**

To compare the health status of one-parent and two-parent families using the McMaster model of family functioning.

**Setting:**

Ondo State, Southwest Nigeria.

**Methods:**

A descriptive cross-sectional design, using multi-stage simple random sampling technique. Data were collected using an adopted self-administered questionnaire from 250 purposely selected families from each sample group. The data entering was analysed using Statistical Package for Social Sciences (SPSS) software version 17.0.

**Results:**

Findings showed that one-parent fathers scored higher (mean = 74.4 ± 10.30) than two-parent fathers (70.5 ± 13.05), while one-parent mothers scored higher (mean = 69.7 ± 15.10) than two-parent mothers (mean 67.7 ± 14.78). This means that one-parent fathers have a better self-reported health status than two-parent fathers, while one-parent mothers have a better self-reported health status than two-parent mothers. One-parent fathers have the best self-reported health status. No significant (*p* > 0.05) difference in the health status of children from both families.

**Conclusion:**

Fathers are healthier than mothers, while one-parent fathers are healthier than two-parent fathers. Comparing the two groups of families, parents from one-parent families reported better health status than parents from two-parent families, whereas within each family group, fathers reported better health status than mothers. This places responsibility on health care professionals to explore family contexts during clinic visits so as to render a more comprehensive health care service to families.

## Introduction

The family is a crucial resource in meeting the needs of and providing care for its members.^1,2^ The family in its various forms, structures and functions is a primary focus of community health; nursing practitioners as a result of the central role it plays in the provision and maintenance of health status of its members and all factors that contribute to achieving optimal health.^1^ Members of the family play different roles but shift in the prevalence of different forms of family. Most importantly, the increase in single-parent households and step families over the past decades underscores the increasing interest on the impact of changing family structure on health status of family members.^3,4^

In the past, two-parent family was regarded as the standard definition viewed by family researchers and policymakers as the model family. It was often used as the yardstick to determine the quality of other families.^5,6^ Although two-parent families are becoming less common, they are still the most common family types around the world. Countries in Asia and the Middle East are usually associated with two-parent families. However, children in America, Europe, Oceania and sub-Saharan Africa are more likely to live in alternative family structures.^7^ A few decades ago, single parenthood or one-parent family was viewed as an aberrant form of the normal family. One-parent form of family was viewed differently from the nuclear family pattern and as such treated as abnormal, and the languages used to describe them were negative. Such languages include; broken families, out-of-wedlock childbearing and father-absence, among others.^4,8^ Previous studies^4,9^ also affirmed that the prevalence of single parenting is increasing consisting of unmarried mothers (which includes teenagers), divorcee and families estranged by migrant work arrangement.

The distribution of family structures varies among countries. There is a steady increase in out-of-wedlock motherhood in sub-Saharan Africa because of marital instability.^10^ Widowhood has been made worse by wars and HIV and AIDS pandemic, leading to increased number of single-mother families in the region.^10,11^ In Nigeria, Salami and Alawode,^12^ over a decade ago, it is commented that the existence of single parents in the country was formerly unknown and where they existed they were ignored as special cases. However, this view has changed over time as they^13^ observed that single-parent family is a fast-growing family pattern in Nigeria. In more recent times, communal clashes, insurgency and terrorism in some parts of Nigeria have contributed to reported increase in the number of single parents and orphans. About 1 million women aged 10–85 years old in Nigeria were either divorced or separated in 2006 and 1.7 million were widowed.^14^

The impact of family structure on the general well-being of family members is well documented in the literature. Single parents were reported to suffer the most with regard to family instability, poverty that affects both parents and their children economically, socially, physically as well as their mental well-being.^2,15,16^ According to Healthy Children,^2^ the day-to-day care-giving, health promotion activities, lifestyle maintenance and family dynamics are complex and they all have influence on health outcome. Consequently, all these have increased the concern on how changes in family structures influence the health and well-being as well as the quality of life for each member of the family. Research has constantly revealed that children living with single-parent families are more likely to experience a diversity of problems than children living with married parents. Children living with single parents have been found to show more emotional,^17^ behavioural^18^ and academic problems^19^ than children living with both of their biological parents and they do less well in series of measures of well-being than their counterparts in two-parent families.^13,17,20,21^

## Theoretical framework

The McMaster model of family functioning (MMFF)^22,23^ is the theoretical framework for this study. The model views the family as an open system that is made up of a complex interplay between different subsystems (individual, marital) that relate to external systems (e.g. extended family, schools, religion and work). The model takes a whole-system approach by evaluating family structure, organisation and transactional pattern.^24^

**FIGURE 1:**
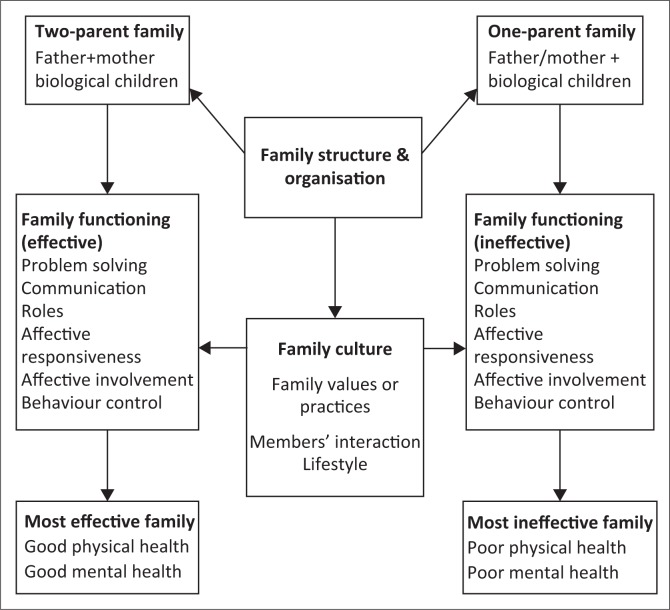
Application of the McMaster model of family functioning to the study.

The MMFF identified various dimensions of family functioning such as; problem solving, communication, roles, affective responsiveness, affective involvement and behavioural control that has impact on the health of family members. The functioning capabilities of a family are based on the culture, values and family practices. The family structure is influenced by certain mediating factors that include; financial resources, parental personal resources, socialisation, stress, time resources as well as the social support available for the family. The conceptual framework for the study according to the MMFF is illustrated in Figure 1. The family assessment device (FAD) is based on MMFF. Families that engage in activities, such as effective problem solving, communicate effectively, perform their roles in the family responsibly, display appropriate feelings over certain issues in the family, are concerned and interested in each other’s activities and concerns, express and maintain standards for the behaviour of the members are said to have effective family functioning.

Two-parent families consists of father, mother and children, and this form of family has a different family structure and organisation when compared with one-parent family made up of either a father or mother and the child or children. Researchers view two-parent families as the golden standard of family structure. It is believed that such families have more efficient and gendered division of labour.^24^ Due to the perceived better economic status of two-parent families, as well as their ability to support or complement one another in family functions, it is expected that they will have better effective family functioning in problem solving, communication, role performance, affective responsiveness, affective involvement and behaviour control within the family.^24,25^ This may be contrary to one-parent families who have only one source of income and are alone in family functioning. Family functioning in both types of families is affected and controlled by family culture, their values and practices, interaction and as well as the lifestyle of family members. The identified subscales for measuring family functioning have direct implications on the health status of family members. Two-parent families have effective family functioning, that will also result in better family health statuses with improved physical, social, mental and emotional health, while one-parent families on the contrary may have ineffective family functioning resulting in reduced family functioning resulting in poor physical, social, mental and emotional health status of the family. The MMFF if consistently applied by health care professionals can result in better effective health care provisions to families. Therefore, the application of the model by various health care professionals working with families will enable them to provide adequate care and support to members from both types of families resulting in better health status and family functioning.

With a documented gap of little focus on families as the clientele in nursing practice in Nigeria,^26^ empirical data on the influence of family structural change and these changes can affect family functioning and consequently the health status of members of the family. Although community health nursing continues to emphasise the family as a unit of service, the health care system encourages individualised services. Consequently, the family is not often considered in treatment plans, interventions to solve health challenges as well as in health programmes. It should also be noted that focus is directed toward individuals in certain age groups or with specific health problems. Hence, the study determines and compares the health status of one-parent and two-parent families in Ondo State, Southwest Nigeria. It is envisaged that the findings of this study will depict the need for family-focused care and provide guides for community health nurses to develop need-based healthcare intervention for families.

## Research methods and design

### Study design

A cross-sectional descriptive design was employed using interviewer-administered questionnaire.

### Setting

Ondo State is located in the southwest geopolitical region of Nigeria with a population of 3.441 million people^27^ and divided into 18 local government areas (LGAs). Culturally, the state inhabits predominantly Yoruba-speaking people and major religions of the people are Christianity, Islam and traditional religion. Data were generated from families drawn from 4 out of the 18 LGAs in Ondo State, Southwest Nigeria.

### Study population and selection of participants

The target population comprised one-parent and two-parent families in the study area. Simple random sampling (SRS) technique was used to select four LGAs from the 18 in the three senatorial districts and to select one-third of the wards in each of the four selected LGAs. Systematic sampling was applied to select households from where two-parent and one-parent families were purposively selected. Criteria for inclusion in the study were as follows, (1) two-parent families with at least one child, (2) one-parent families, which can be either the father or the mother with at least one child and (3) willingness to participate in the study. The sample size was determined using sample size formula for comparing proportions and means of independent groups. The sample size was 250 families for each of the two groups. Multi-stage sampling technique was used to select participants for the study. At the first stage, two out of the three senatorial districts in Ondo State were selected by SRS, while at the second stage, two local governments were selected from each selected senatorial district by SRS, and at the third stage, one-third of the ward in each selected local government were selected by SRS. At the fourth stage, 50% of streets from each ward were selected by SRS. At the fifth stage, households were selected by systematic sampling, the required numbers of two-parent families were selected from the household until the required sample size was reached, and the first household on the selected street was the first point of selecting families after which every fourth house was selected. Both parents in a two-parent family whose one of their children’s age is between 10 and 17 years were selected. A purposive selection of the required number of one-parent families on each street was performed. One-parent families, either male or female with one of the children between 10 and 17 years, were selected.

### Instrument

Data were collected using interviewer-administered questionnaire using English and Yoruba versions of the instrument. Two experts who are Yoruba linguists confirmed the adequacy of the translation when compared with the English version evaluated post-translation content validity. Test–retest was also carried out during pilot study, coefficient of FAD is 0.86, SF12 0.80 and CHQ 0.94. Firstly, an adopted Rand 12-item Health Survey questionnaire (SF-12)^28^ was used to measure parents’ health status. Test items on the questionnaire were grouped into eight principal health domains that included physical functioning, role limitation because of physical health, role limitation because of emotional problems, energy or fatigue, emotional well-being, social well-being pain and general health, and the variables were measured using Likert scale. Then, an adapted form of child health questionnaire 87 (child form)^29^ was used to measure child’s health status. All the test items on the questionnaire were arranged into nine principal health domains that are: physical functioning, role functioning (emotional), role functioning (behavioural), role functioning (physical), bodily pain, mental health, self-esteem, general health perception and change in health. All questions were scored on a scale of 0–100, with 100 representing the highest level of health status possible. A higher score indicates more favourable ratings of health and well-being. Each of the questionnaires was in two sections. Section A contained the socio-demographic characteristics of respondents, while section B contained questions to assess their health status. The validity of instruments was established through face and content validity by ensuring that test items covered every aspect of the study. The questionnaire was given to scholars in the field of community health nursing for proper scrutiny and correction. The questionnaire was pilot-tested using participants who had similar characteristics with the study participants but the data were not included in the results.

FDA reliability has been documented in several studies with a range of alphas between 0.72 and 0.92, internal consistency reliability was high at 0.89 and validity also high at 0.78.^30^ The interviewer-administered questionnaire contains 12 items of the general functioning on the FAD which measures family functioning based on the following set of subscales: problem solving, communication, roles, affective responsiveness, affective involvement and behaviour control. The variables were measured using Likert scale at four levels of: strongly agree, agree, disagree and strongly disagree. Family member’s responses were scored for each sub-scale; FAD is scored by summing the responses (1–4) for each sub-scale and dividing by the number of items in each scale and mean family scores were determined. These were compared with set cut-off scores for each sub-scale on the MMFF scale to determine the functionality of the families. A higher score represents higher family functioning.

### Data collection

Data were collected over a period of three months by the first author and two research assistants. Research assistants were recruited and trained for one week, and the first author and research assistants contacted respondents through home visits at prearranged time; revisits were made where necessary in order to meet family members. Further briefing on the study aims and objectives was done prior to the participants completing and signing the consent forms. The father, mother and one child, were interviewed in two-parent families, but the father or mother and a child in one-parent families were interviewed (where families had more than one child, availability was considered and where necessary simple randomisation was performed). Interviews with each member of the family were conducted privately and individual information was not shared. A Yoruba version of the instrument was used where necessary.

### Data analysis

Data entering was performed by Statistical Package for Social Sciences (SPSS) software version 17.0 using both descriptive and inferential statistics. Health statuses of two-parents and one-parent families were determined and compared. The parents’ and child’s health were measured using mean score and standard deviation. The differences between the health status of both parents and children from two-parent and one-parent families were calculated using *t*-test, while differences between health statuses of all family members were calculated using analysis of variance (ANOVA).

## Ethical considerations

The ethical clearance for the study was granted by the Health Research Ethics Committee of the Institute of Public Health of the College of Health Sciences, Obafemi Awolowo University, Ile-Ife (IPH/OAU/12/273). Approval was also granted by the community leaders in each of the local government areas (LGAs). The purpose of the study was explained to each participant and both verbal and written consent were obtained from respondents prior to data collection. Confidentiality was ensured by using codes for each questionnaire and completed questionnaires were kept in locked drawers to ensure limited access to information by people unauthorised and not part of the research team. The investigator educated, counselled and referred respondents after the completion of the study according to the needs to promote their health and help them seek care as desirable based on the results of their health status assessment.

## Results

### Demographic profile

The demographic data of the participants from two-parent and one-parent families are summarised in Table 1. A total of 250 fathers and 250 mothers from two-parent families participated in the study. While there are more mothers (211 = 84.4%) than fathers (39 = 15.6%) in one-parent families, the mean age of parents was 45 years. The mean monthly family income was N74 000 (about $250).

**TABLE 1:** Demographic characteristic of participants (parents).

Characteristics	Two-parent families (*n* = 500)		One-parent families (*n* = 250)	
	Frequency	%	Frequency	%
**Gender**				
Male	250	50.0	39	15.6
Female	250	50.0	211	84.4
**Age in years**				
20–40	216	43.2	100	40.0
41–60	236	47.2	120	48.0
Above 60	48	9.6	30	12.0
**Educational status**				
No formal education	30	6.0	13	5.2
Primary	110	22.0	58	23.2
Secondary	240	48.0	105	42.0
Tertiary	120	24.0	74	29.6
**Family monthly income**				
Up to $60	50	20.0	94	37.6
$61–$135	62	24.8	76	30.4
$136–$270	100	40.0	75	30.0
Above $271	38	15.2	5	2.0

The demographic data of children from two-parent and one-parent families are summarised in Table 2. There are 250 children from each family. The mean age of children is 14 years, and almost all (99.7%) are still schooling.

**TABLE 2:** Demographic characteristic of children.

Characteristics	Two-parent families (*n* = 250)		One-parent families (*n* = 250)	
	Frequency	%	Frequency	%
**Gender**				
Male	91	36.4	48	19.2
Female	159	63.6	202	84.4
**Age in years**				
10–13	161	64.7	148	59.2
14–17	89	35.3	102	40.8
**Educational status**				
No formal education	1	0.3	-	-
Primary	51	21.7	72	28.8
Secondary	190	75.2	175	70.0
Tertiary	8	2.8	3	1.2

### Data on fathers’ health status

The breakdown of information on the health status of fathers from one-parent and two-parent families on each of the eight health domains is summarised in Table 3. Fathers from one-parent family had higher mean scores than fathers from two-parent family in all the domains except in physical functioning (one-parent fathers, mean = 80.7 ± 28.94; two-parent fathers, mean = 81.6 ± 28.40) and general health (one-parent fathers, mean = 80.1 ± 18.20; two-parent fathers, mean = 83.2 ± 19.30), and there are no statistical differences in the mean scores across the two groups. Equally, fathers from one-parent family had a higher mean score in pain than two-parent family (one-parent fathers mean = 67.9 ± 26.25; two-parent fathers mean = 65.0 ± 23.11 with *p* < 0.05).

**TABLE 3:** Health status of fathers using the eight health domains.

Health status domains	Fathers						
	One-parent (*n* = 39)		Two-parent (*n* = 250)		*t*	*p*
	Mean	s.d.	Mean	s.d.		
Physical functioning	80.7	28.94	81.6	28.40	0.394	0.644
Role limitation (physical health)	94.1	19.18	88.1	28.07	−0.557	0.578
Role limitation (emotional problem)	94.8	19.18	85.3	31.53	0.413	0.680
Energy or fatigue	82.6	26.03	78.6	25.43	1.082	0.280
Emotional well-being	81.3	21.42	80.0	19.28	0.749	0.454
Social functioning	86.5	20.56	80.9	23.19	−0.785	0.433
Pain	67.9	26.25	65.0	23.11	2.868	0.004
General health	80.1	18.20	83.2	19.30	−0.946	0.348

s.d., standard deviation.

### Data on mothers’ health status

Data on health status of mothers from one-parent and two-parent families on each of the health domains are presented in Table 4. Mothers from one-parent families scored higher in all the domains except in role limitation because of physical health (one-parent mothers, mean = 84.6 ± 32.91, two-parent mothers, mean = 86.2 ± 30.37), social functioning (one-parent mothers, mean = 79.4 ± 23.56; two-parent mothers, mean = 81.1 ± 24.91) and general health (one-parent mothers mean = 81.7 ± 19.0; two-parent mothers’ mean = 84.0 ± 18.9).

**TABLE 4:** Health status of mothers using the eight health domains.

Health status domains	Mothers						
	One-parent (*n* = 211)		Two-parent (*n* = 250)		*t*	*p*
	Mean	s.d.	Mean	s.d.		
Physical functioning	80.1	32.01	79.0	28.51	−0.180	0.857
Role limitation (physical health)	84.6	32.91	86.2	30.37	1.457	0.146
Role limitation (emotional problem)	83.4	32.87	82.2	33.46	1.845	0.066
Energy or fatigue	74.1	28.68	71.1	31.93	0.910	0.036
Emotional well-being	79.9	21.55	78.6	19.48	0.379	0.705
Social functioning	79.4	23.56	81.1	24.91	1.434	0.147
Pain	68.4	24.47	61.9	25.19	0.835	0.404
General health	81.7	19.00	84.0	18.90	−1.306	0.192

s.d.,standard deviation.

### Overall health status of parents from one-parent and two-parent families

Table 5 compares the overall health status of parents from one-parent and two-parent families. One-parent fathers had the highest score (mean = 74.4.0 ± 10.30), followed by two-parent fathers (mean = 70.5 ± 13.05) and one-parent mothers (mean = 69.7 ±15.10), while the two-parent mothers (mean = 67.7 ± 14.78) had the least health status.

**TABLE 5:** Comparison of overall health status of parent from one-parent and two-parent families.

Health status of parents	Fathers				Mothers				*df*	*F*	Sig.
	One-parent (*n* = 39)		Two-parent (*n* = 250)		One-parent (*n* = 211)		Two-parent (*n* = 250)				
	Mean	s.d.	Mean	s.d.	Mean	s.d.	Mean	s.d.			
Overall health status	74.4	10.30	70.5	13.05	69.7	15.10	67.7	14.78	3	3.722	0.011

s.d., standard deviation; *df*, degrees of freedom; Sig., significance.

### Data on children’s health status

Health status of children from one-parent and two-parent families on each of the health status domains is summarised in Table 6. The findings revealed that mean scores of the children in both families in all the domains were very similar. One-parent children had lower scores in self-esteem and role limitation because of emotional problems. For self-esteem, one-parent children had a mean of 76.9 ± 14.45 and two-parent children had a mean of 79.3 ± 12.70, while for role limitation because of emotional problems, one-parent children had a mean of 93.3 ± 21.46 and two-parent children had a mean of 95.0 ± 18.23.

**TABLE 6:** Health status of children using the eight health domains.

Health status domains	One-parent families (*n* = 250)		Two-parent families (*n* = 250)		*t*	*p*
	Mean	s.d.	Mean	s.d.		
Physical functioning	93.5	19.28	94.8	16.74	0.749	0.454
Role functioning (emotional	93.3	21.46	95.0	18.23	0.947	0.344
Role functioning (behavioural)	94.9	18.85	95.7	16.70	0.494	0.621
Role functioning (physical)	93.0	21.41	93.7	19.64	0.334	0.739
Bodily pain	76.8	16.46	76.9	17.89	0.121	0.904
Mental health	80.2	12.56	80.1	12.06	−0.36	0.972
Self esteem	76.9	14.45	79.3	12.70	1.626	0.105
General health perception	68.7	8.86	68.2	10.30	−0.522	0.602
Change in health	77.5	20.97	78.0	21.61	0.268	0.548

s.d., standard deviation.

### Overall health status of children from one-parent and two-parent families

The comparison of health status of children from one-parent and two-parent families is summarised in Table 7. The mean scores of children from one-parent family are similar but not significantly different from the scores of children from two-parent family (84.8 ± 9.47 vs. 84.4 ± 9.67; *p* > 0.05).

**TABLE 7:** Comparison of overall health status of children from one-parent and two-parent families.

Overall Health status	One-parent families (*n* = 250)		Two-parent families (*n* = 250)		*t*	*p*
	Mean	s.d.	Mean	s.d.		
Comparison	84.8	9.47	84.4	9.67	−0.484	0.628

s.d., standard deviation.

## Discussion

As shown in the study, parents and children from both families have similar demographic characteristics, although the average monthly family income of one-parent family is lower than the monthly family income of two-parent family. This is consistent with findings from previous studies^6,31,32,33^ that have associated single parenthood with more financial burden compared to two-parent families.

As revealed in this study, there are few (15.6%) single-parent fathers when compared to single-parent mothers (84.4%). This study supports earlier observation by Bramlet and Blumberg^20^ that in spite of increase in the prevalence of one-parent family, there are more one-parent mothers than one-parent fathers. Similarly, Casper et al.^34^ stated in their study that children often reside with mothers following family instability and that one-parent father’s status is often not permanent as they usually cohabit or remarry early unlike one-parent mothers. Women tend to concentrate on bringing up their children much longer before considering the option of remarrying or otherwise. Nurses need to take cognisance of this challenge in the context of caring and giving more support to single mothers and single fathers who are not in new relationships, especially watching out for possibility of burnout.

The study further reveals that one-parent fathers have better health status than two-parent fathers in all the domains except in physical functioning and general health. Single fathers perform home activities that are meant for both father and mother and need to also cope with work. This may be the reason for poor physical functioning, which eventually may lead to poor general health. However, this is not reflected in energy or fatigue and other domains of health.

Although previous studies have reported that single fathers, who live with their children, report poorer health than cohabiting fathers,^35,36^ another study reported that non-custodial fathers had higher mortality risks than custodial fathers.^37^ This may be as a result of the absence of a partner to relate with, which may increase psychological distress and result in poor health. Equally, Umberson^38^ stated that divorced fathers, who live with their children, engage in less dangerous health behaviour when compared with divorced fathers who live without their children. One-parent mothers in comparison with two-parent mothers also had poorer health in certain areas, which include role limitation because of physical health, general health and social functioning. One-parent mothers bear more of the household and parental responsibilities compared to their married counterparts. In addition to workload, there is a suggestion that the network of resources that parents can access through their activities in life outside the family contributes in some way to their physical and psychological well-being.^2,39^ Fewer resources in terms of time, money and social networks accruable to one-parent mothers may be responsible for this health outcome that may result in physical and psychosocial stress with further negative implications on health and well-being.^6,40,41,42^ This finding confirms the need to give extra attention to single-parent family in the aspect of helping to promote optimal family functioning. Nurses must essentially assess for family functioning, adopt family-centred care before making appropriate nursing diagnoses and develop nursing care plan that would be comprehensive to meet family needs. It is also advocated even as health care needs of individuals become the source of contact with the health care system.

Social functioning means the ability to develop and maintain social relationships and the ability to carry out roles as members of a household or social group. Poor social functioning may be attributed to some level of stigma attached to single parenthood in some African culture^13^ and this may predispose single mothers to poor emotional health. Fathers impact positively on their wives by providing emotional support for mothers in two-parent households compared to single parents who have been reported to experience more periods of depression and higher degree of stress than married mothers.^43^ In this study, two-parent mothers had low scores in the domain of pain and energy or fatigue compared with one-parent mothers. With changing roles, women have become a noticeable part of the workforce.^43,44^ They not only care for their husbands, children and home, but also engage in other activities that bring income into the family. Previous studies stated that two-parent mothers have better health than single-parent mothers,^43,44.45,46^ possibly because marriage and cohabitation have a protective effect that arises from improved material resources. In addition to improving the socio-economic status, marriage guards against dangerous negative health behaviours, susceptibility to lack of social networks and a lack of social support.^40^

Further result in this study showed that the scores of children from two-parent and one-parent families in all health domains are similar, signifying the same level of health status. However, this is not consistent with findings of earlier studies that indicated that children from one-parent households have worst health status when compared with their counterpart from two-parent households.^4,17^ Besides, it was observed in this study that children from one-parent households had poor health in self-esteem and role limitation, which is consistent with the findings of previous studies^47^; that boys from married homes have higher self-esteem compared with boys from one-parent homes. Marital separation commonly involves major emotional distress for children, predisposing children from one-parent family to poor self-esteem and emotional problems.^13,20,44,48^

A review of evidence on the implication of family structure on the health of family members, especially children, found from ‘serious’ to ‘no effect’, noting that differences in health status among children in two-parent and single-parent homes are not a result of structure but the economic status.^17,44^ Therefore, children from single parents who have good economic status and are educated may not have poor health.^17^ Although lower social-economic factor is a major cause of poor health outcomes for individuals,^49^ families with poor socio-economic status irrespective of its structure are likely to be presented with poor health outcomes. Other considerations should include the degree of family structure and available support for families, indicating that families with poor socio-economic status and poor support system, irrespective of family structure, will encounter poor health status, whereas if there are adequate social and family supports to cushion the poor socio-economic status, there may not be negative health effects.

## Limitations

The purposive sampling methods limit the generalisation of study results to a larger context. Also, the study was a cross-sectional design, which precludes a cause and effect conclusion. The low number of one-parent fathers may also have influenced the study findings by under-powering it to detect significant differences between mothers and fathers. In addition, all the instruments used in this study are self-reported which has its own shortcomings.

## Conclusion and recommendations

Findings from this study have shown that parents from one-parent families have better health status than parents from two-parent families. The type of family structure appears not to have differential effects on the health status of family members except in the domain of pain and fatigue. While this places responsibility on health care professionals to explore family contexts during clinic visits, there is a need to develop policies that target one-parent families (especially women) for socio-economic and mental health support.

However, it is recommended that a study that is more extensive should be conducted on this subject in other parts of Nigeria. It is also recommended that the focus should be on the implication of single parenthood on health, socio-economic status and the family support system and in order to give a generalised knowledge about the health status of families in relation to their structure in Nigeria.
